# A *ΔclpB* Mutant of *Francisella tularensis* Subspecies *holarctica* Strain, FSC200, Is a More Effective Live Vaccine than *F. tularensis* LVS in a Mouse Respiratory Challenge Model of Tularemia

**DOI:** 10.1371/journal.pone.0078671

**Published:** 2013-11-13

**Authors:** Igor Golovliov, Susan M. Twine, Hua Shen, Anders Sjostedt, Wayne Conlan

**Affiliations:** 1 National Research Council Canada, Human Health and Therapeutics Portfolio, Ottawa, Ontario, Canada; 2 Department of Clinical Microbiology, Clinical Bacteriology, Umeå University, Umeå, Sweden; The Scripps Research Institute and Sorrento Therapeutics, Inc., United States of America

## Abstract

*Francisella tularensis* subsp. *tularensis* is a highly virulent pathogen for humans especially if inhaled. Consequently, it is considered to be a potential biothreat agent. An experimental vaccine, *F. tularensis* live vaccine strain, derived from the less virulent subsp. *holarctica*, was developed more than 50 years ago, but remains unlicensed. Previously, we developed a novel live vaccine strain, by deleting the chaperonin *clpB* gene from *F. tularensis* subsp. *tularensis* strain, SCHU S4. SCHU S4*ΔclpB* was less virulent for mice than LVS and a more effective vaccine against respiratory challenge with wild type SCHU S4. In the current study, we were interested to determine whether a similar mutant on the less virulent subsp. *holarctica* background would also outperform LVS in terms of safety and efficacy. To this end, *clpB* was deleted from clinical *holarctica* strain, FSC200. FSC*200ΔclpB* had a significantly higher intranasal LD_50_ than LVS for BALB/c mice, but replicated to higher numbers at foci of infection after dermal inoculation. Moreover, FSC*200ΔclpB* killed SCID mice more rapidly than LVS. However, dermal vaccination of BALB/c mice with the former versus the latter induced greater protection against respiratory challenge with SCHU S4. This increased efficacy was associated with enhanced production of pulmonary IL-17 after SCHU S4 challenge.

## Introduction

*Francisella tularensis* is a facultative intracellular bacterial pathogen of mammals. Two subspecies, subsp. *holarctica* and subsp. *tularensis* are highly infectious for humans, and the latter can cause high mortality when inhaled [1]. Consequently, subsp. *tularensis* was developed as a biological weapon by several countries during the 20^th^ century [2]. Tularemia caused by subsps. *holarctica* was endemic in the USSR during the early 20^th^ century. To counter this threat, Soviet scientists developed an attenuated *holarctica* strain, strain 15, as a live vaccine. This vaccine was highly effective at preventing tularemia caused by subsp. *holarctica* [3]. However, its efficacy against subsp. *tularensis* was not assessed. Subsequently, the USA developed a phenotypically more defined live vaccine strain (LVS) from strain 15 [4]. LVS was tested extensively in human volunteers during the 1960s, and was shown to initially provide 80–90% protection that waned over time against substantial intradermal or inhaled inocula of subsp. *tularensis* strain, SCHU S4 [5], [6]. For various regulatory reasons, LVS has never been licensed for public use [7]. Nevertheless, it is currently being reassessed for safety and immunogenicity, but not efficacy, in several clinical trials (http://www.clinicaltrials.gov/ct2/show/NCT01150695?term=tularemia&rank=1).

We and others have been investigating a variety of approaches to develop more defined, and more effective tularemia vaccines using murine models of respiratory infection with SCHU S4 as a preclinical screen (reviewed in [8]). Several of us, have focussed on devising defined attenuated mutant strains of SCHU S4 for use as live vaccines on the grounds that they contain antigens unique to subsp. *tularensis* that could be important targets for the protective immune response [9]–[11]. Several such mutants have been shown to be highly effective. In our hands, SCHU S4*ΔclpB* has performed well in this respect [12]–[15].

A potential concern about SCHU S4 deletion mutants is their ability to revert to a virulent phenotype. In this regard, it has recently been shown that LVS is attenuated almost entirely because of partial deletion of a single gene [16]. Moreover, strain 15, the parent strain of LVS was given to millions of Russians with no documented evidence of its reversion [3]. Nevertheless, a defined vaccine strain on a subsp *holarctica* background could help alleviate any lingering concerns, since it, like LVS, could at most revert to the less virulent subsp. *holarctica* phenotype. To determine whether such a strain remains an effective vaccine, we have deleted the *clpB* gene from a fully virulent subsp *holarctica* strain, FSC200.

Compared to LVS, FSC200*ΔclpB* replicated to greater numbers in target organs, but was less lethal than the former via the respiratory route. As with SCHU S4Δ*clpB*, ID immunization of BALB/c mice with FSC200Δ*clpB* elicited greater protection than LVS against respiratory challenge with SCHU S4. Additionally, like SCHU S4Δ*clpB,* enhanced protection by FSC200Δ*clpB* versus LVS was associated with increased levels of IL-17 in the lungs after challenge with virulent bacteria. However, in a head-to-head comparison, FSC200Δ*clpB* was less effective than SCHU S4Δ*clpB*. Furthermore, FSC200*ΔclpB* killed severely immunocompromised SCID mice more rapidly than either SCHU S4Δ*clpB* or LVS.

## Materials and Methods

### Bacterial Strains

*F. tularensis* subsp. *tularensis* strain SCHU S4, and subsp. *holarctica* strain FSC200, were obtained from the Francisella Strain Collection, Umea University, Sweden. Both are clinical isolates, and both are highly virulent for mice (LD_100_<10 CFU) [16], [17]. The *ΔclpB* deletion mutants of both strains were prepared by allelic replacement resulting in mutants with no foreign DNA as previously described [18]. Complementation was performed as we previously described [19]. LVS was a low passage stock prepared from NDBR lot #11 [20].

### Mice & Ethics

Specific pathogen free BALB/c mice and BALB/c SCID mice were from Charles Rivers Laboratories, St. Constant, Que. Animal experiments described herein were approved by the National Research Council (NRC) Animal Care Committee (ACC) and conducted in a Canadian Council on Animal Care (CCAC) accredited facility. Mice were examined daily for signs of infection. Whenever feasible, mice were euthanized by CO_2_ asphyxiation as soon as they displayed signs of irreversible morbidity. In our experience, such mice were at most 24 h from death, and the time to death of these animals was estimated on this premise. All work with SCHU S4, FSC200, andmutants thereof was performed in a small animal biocontainment level 3 facility certified by the Canadian Food Inspection Agency and the Public Health Agency of Canada.

### Immunization and Challenge

Mice were vaccinated with various doses of one or other of the vaccine strains administered intradermally (ID) in a volume of 50 µl of saline, or intranasally (IN) in 20 µl then chased with 20 µl sterile saline. On the stated days post-vaccination, some mice were killed and skin, spleen, lung, and serum assessed for bacterial levels as described previously [14]. Additionally, some immunized mice were bled on day 28 post-vaccination and their sera used for immunoproteomics. Finally, representative mice from each group were challenged IN six weeks post-vaccination with wild-type SCHU S4 and either killed at intervals for pulmonary cytokine analysis or monitored for survival.

### Cytokines and Chemokines

Lung homogenates were clarified by centrifugation and 0.22 µm filtration, and confirmed to be sterile by plating. Levels of pulmonary cytokines and chemokines in these samples were determined using Fluorokine Mouse 21-plex Cytokine Detection System (R & D systems, Minneapolis, MN) on a Luminex® 100 IS system (Luminex, Austin, TX). Cytokine/chemokine concentrations were calculated against the standards using Beadview® software version 1.03.

### Immunoproteomics

A total cell lysate of *F. tularensis* SCHU S4 was used as antigen for immunoproteomics studies as described in our recent work [14]. Proteins were separated in the pH range 4–7 using a linear gradient and then using a 12% SDS-PAGE. Proteins separated by 2D PAGE were electroblotted onto PDVF membranes and probed with murine sera at a dilution of 1∶500, chosen on the basis of previous work. Reactive spots were visualized using the ECL Chemilumiscence kit (GE Life sciences USA). PDQuest software (Biorad, USA), was used to align protein stained gel images of with images of the developed blots. Immunoreactive spots were excised from matched protein stained 2D-PAGE gels and tryptically digested prior to analysis by nano-liquid chromatography nLC-MS/MS exactly as described in earlier work [14].

### Statistics

Statistical analyses were performed with GraphPad Prizm version 6.0 software. Bacterial burdens and cytokine levels were compared by t-test on log transformed data. Survival curves were compared by Mantel-Cox test with Bonferroni correction as appropriate. In all cases, a corrected P<0.05 was considered statistically significant.

## Results

### Virulence of FSC200Δ*clpB* Versus LVS for BALB/c Mice

**Figure 1:**
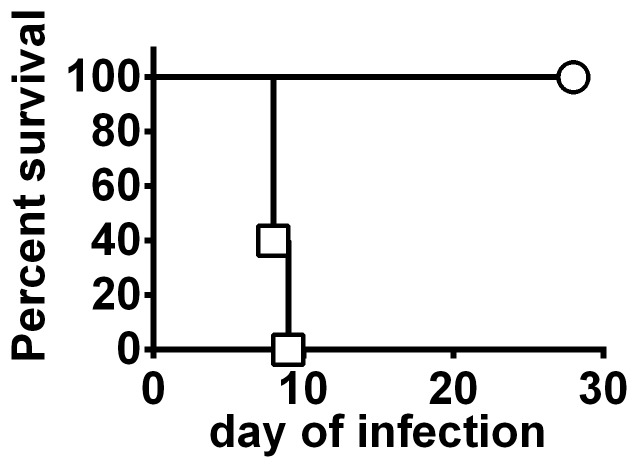
Attenuation of FSC200*ΔclpB* for mice. BALB/c mice (n = 5/group) were inoculated ID with 10^7^ CFU of FSC200*ΔclpB* (circle) or 100 CFU of the complemented strain (square) and monitored for survival.

**Figure 2:**
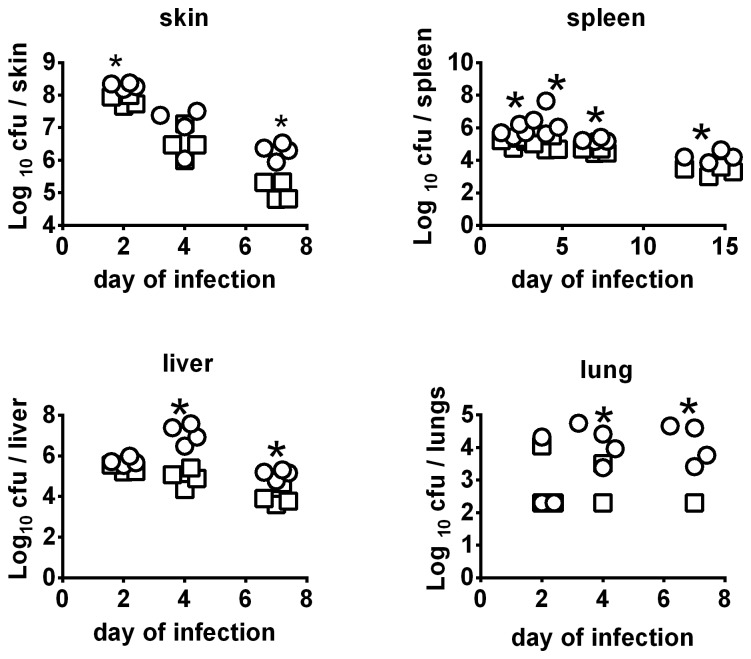
*In vivo* growth kinetics of FSC200 *ΔclpB* vs LVS following ID vaccination. BALB/c mice (n = 4/group) were immunized ID with ∼10^5^ CFU of FSC200 *ΔclpB* (circle) or LVS (square). Mice were killed on days 2,4,7,and 14 for bacteriology. *, significantly higher burden vs LVS. Symbols not shown for organ burdens below detectable limits (100 CFU).

Deleting the *clpB* gene from FSC200 created a highly attenuated strain and complementation restored virulence (figure 1 & figure S1 in File S1). LVS is also highly attenuated by the ID route, however it retains substantial virulence for mice via the IN route and was found to be significantly more lethal than FSC200*ΔclpB* in this regard (figure S2 in File S1). The *in vivo* growth of an ID inoculum of 10^5^ CFU FSC200*ΔclpB* compared to LVS is shown by figure 2. By day 2, there had been a mean 630-fold versus 1600- fold increase in burden of LVS versus FSC200Δ*clpB* at the site of inoculation (P<0.05). Bacterial burdens declined in the skin in both groups on days 4 and 7, but FSC200Δ*clpB* persisted at a significantly higher level than LVS at the latter time-point. The skin was sterile in both groups by day 14 post inoculation. Both strains had disseminated to the spleen by day 2, and persisted therein for at least 14 days. At all time-points, FSC*200*Δ*clpB* was present at significantly higher levels than LVS in this organ. A similar situation was observed in the liver on days 4 and 7 after infection, but both strains were cleared from this organ by day 14. In the lungs, LVS and FSC200Δ*clpB* were recovered from 3/4 mice on day 2, but the former was recovered only sporadically thereafter compared to the latter. Both strains had been cleared from the lungs by day 14. In the blood, 1 or 2 colonies (100–200 CFU/ml), were recovered from 1 or 2 mice on days 2 and 4 after vaccination. A similar result was obtained upon repeat (figure S3 in File S1).

During the course of this experiment, most immunized mice remained completely asymptomatic except that one mouse immunized with LVS died on day 4, and a few others in both groups displayed mild pilo-erection between days 5 and 6. Additionally, using the skin reaction scale shown in figure S4 in File S1, only 3/25 mice immunized with FSC200*ΔclpB* reached a score of 2 compared with 19/25 mice immunized with LVS.

### Efficacy of ID Vaccination with FSC200Δ*clpB* Versus LVS Against Respiratory Challenge with SCHU S4

**Figure 3:**
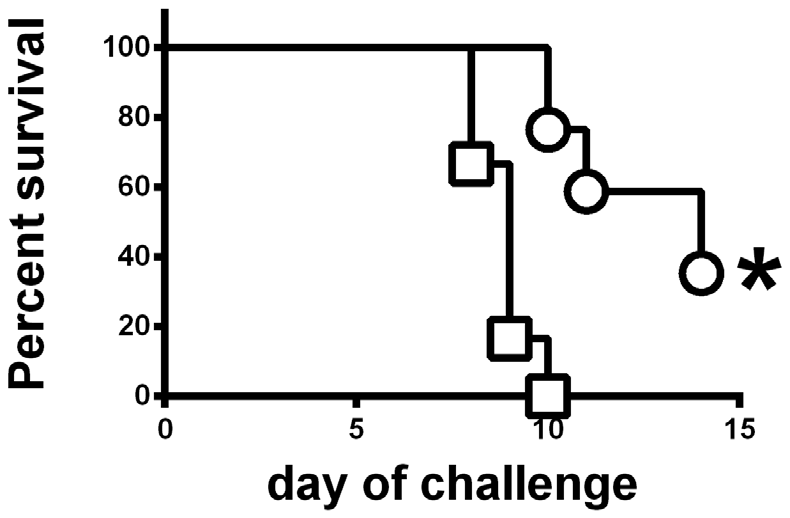
Protective efficacy of ID vaccination with FSC200*ΔclpB versus* LVS against respiratory challenge with SCHU S4. BALB/c mice were immunized ID with 10^5^ CFU of LVS (square; n = 12) or FSC200*ΔclpB* (circle; n = 17) and challenged 6 weeks later IN with 86 CFU of SCHU S4. *, Significantly longer survival than mice immunized with LVS.

**Figure 4:**
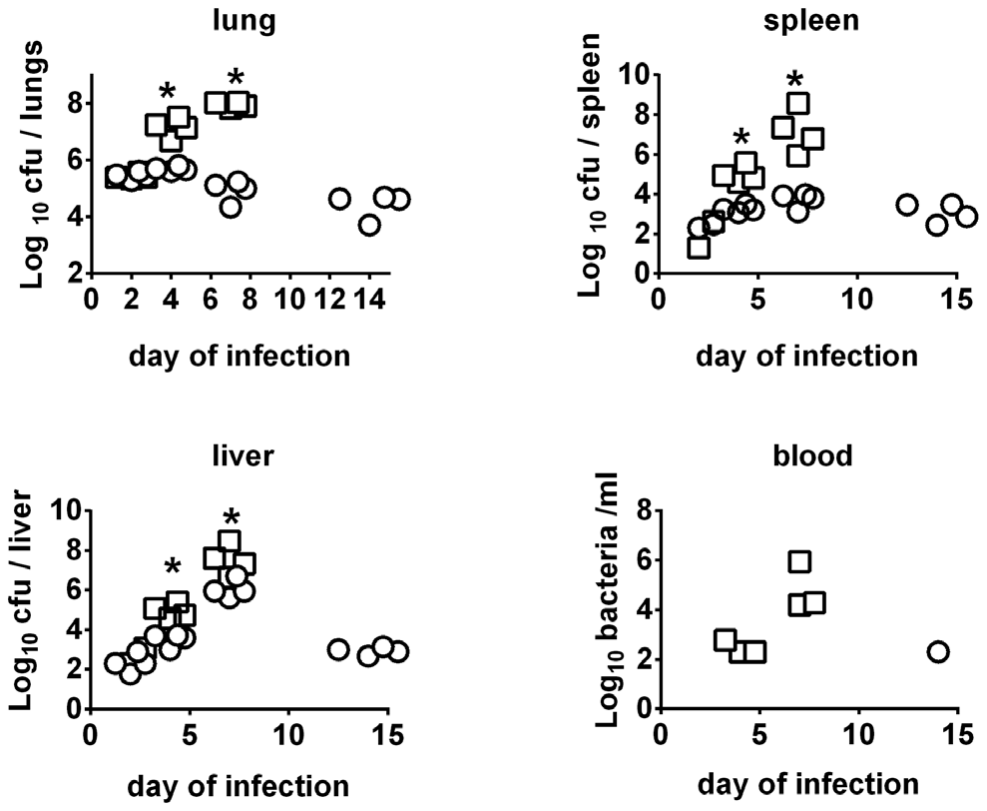
Kinetics of SCHU S4 infection in mice immunized with FSC200 *ΔclpB* vs LVS. BALB/c mice (n = 4/group) were immunized ID with ∼10^5^ CFU of FSC200 *ΔclpB* (circle) or LVS (square). Six weeks later immunized and control mice were challenged IN with 86 CFU of SCHU S4. Mice were killed on days 2,4,7,14 after challenge and bacteriology performed on their organs. *, significantly higher burden vs mice immunized with FSC200 *ΔclpB*.

LVS is only approved for use in humans by the scarification method. To mimic this situation, and to allow more precise quantification of the inoculating dose, mice were immunized by the ID route. Six weeks after ID immunization with ∼10^5^ CFU of FSC200Δ*clpB* or LVS, mice were challenged IN with 86 CFU of SCHU S4. Some mice were killed on days 2, 4, 7, and 14 and organs and sera examined for bacteria, and the lungs for cytokines, and chemokines. All naïve mice died on day 5 after challenge, no mice immunized with LVS survived past day 10, and some mice immunized with FSC200Δ*clpB* died between days 10–14 (figure 3) ). By day 2 there had been obvious growth of SCHU S4 in the lungs of mice immunized with either LVS or FSC200Δ*clpB* and it continued to grow in the lungs of the former mice until they succumbed to infection between days 8 and 10 after challenge (figure 4). In contrast, after day 2, surviving mice immunized with FSC200Δ*clpB* were able to control SCHU S4 infection in the lungs, but not eliminate it during the ensuing twelve day observation period. A similar situation was seen in the spleen, whereas in the liver SCHU S4 grew up to day 7 in both groups of mice. Thereafter, infection was brought under control only by the mice immunized with FSC200Δ*clpB*. Bacteria were recovered from the blood of most mice immunized with LVS on days 4 and 7 after challenge with SCHU S4, but only from the blood of a single mouse immunized with FSC200Δ*clpB*.

### Cytokine and Chemokine Responses in Vaccinated Mice Following IN Challenge with SCHU S4

**Figure 5:**
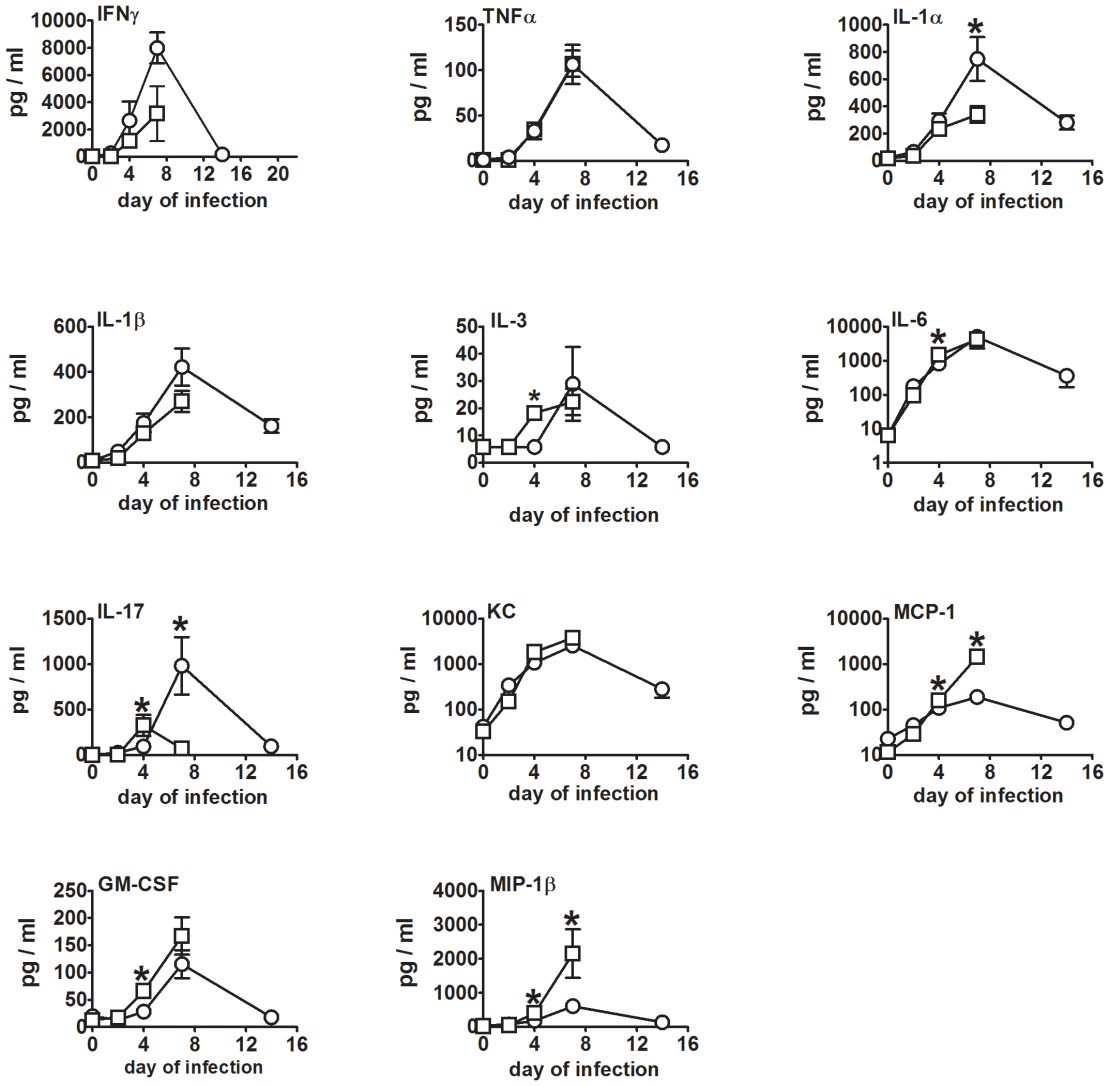
Kinetics of pulmonary cytokine production after challenge of vaccinated mice with SCHU S4. BALB/c mice (n = 4/group) were immunized ID with ∼10^5^ CFU of FSC200 *ΔclpB* (circle) or LVS (square). Six weeks later immunized mice were challenged IN with 86 CFU of SCHU S4. Mice were killed on days 2,4,7,14 after challenge and Luminex performed on their lungs. *, significantly higher level vs other vaccinated group.

Previously, we showed that in BALB/c mice, vaccination with SCHUΔ*clpB* was more effective against respiratory challenge with wild-type bacteria than vaccination with LVS [13]. Additionally, we showed that SCHUΔ*clpB* protects BALB/c mice but not C57BL/6 mice against such challenge [14]. In both cases, the enhanced efficacy of SCHU*ΔclpB* was associated with significantly higher levels of IL-17 in the lungs seven days after challenge with SCHU S4. To determine if this was the case too for FSC200Δ*clpB* versus LVS, we examined the lungs of vaccinated mice challenged IN with SCHU S4 for a panel of cytokines and chemokines (Figure 5). For the most part, cytokine and chemokine levels rose steadily over the course of the first 7 days of challenge in both groups, then declined between day 7 and 14 in surviving mice. The exceptions were IL-17 which peaked on day 4 in mice immunized with LVS, and IL-5, IL-12p40, IL-12p70, IL-13, and RANTES which remained at or close to background throughout.

By day 2 after challenge with SCHU S4, pulmonary IL-6 and KC were significantly elevated over background levels in both groups of immunized mice, whilst IFNγ, IL-1α, IL-1β, and IL-17 levels were significantly elevated only in mice immunized with FSC200Δ*clpB*; MCP-1 was significantly elevated only in LVS-immunized mice. By day 4 after challenge with SCHU S4 pulmonary levels of IFNγ, IL-1α, IL-1β, IL-6, IL-17, KC, MCP-1, MIP-1β, and TNFα, were significantly above background in both groups of immunized mice, whereas IL-3 and GM-CSF were significantly elevated only in mice vaccinated with LVS. The situation was the same on day 7 after challenge, except than GM-CSF and IL-3 were now significantly elevated in both groups. No mice immunized with LVS survived to day 14 after challenge with SCHU S4, at which point most cytokines and chemokines had dropped from day 7 levels in mice immunized with FSC200Δ*clpB*. However, IFNγ, IL-1α, IL-1β, IL-6, IL-17, KC, MCP-1, MIP-1β, and TNFα were still significantly above background levels at this time. We have observed similar results for LVS and SCHU S4 Δ*clpB* in previous studies [13], [14].

A comparison of pulmonary cytokine and chemokine levels between the two vaccinated groups of mice showed that MCP-1, MIP-1β, GM-CSF, and IL-17 levels were significantly higher in the lungs of mice immunized with LVS versus FSC200*ΔclpB* on day 4 after challenge with SCHU S4 as were the former two cytokines on day 7. However, IL-17 and IL-1α levels were significantly higher in mice immunized with FSC200*ΔclpB* on day 7 after challenge with SCHU S4. We have obtained similar results for IL-17 in BALB/c mice immunized with SCHU S4*ΔclpB* [13].

### Immunoproteomics

**Figure 6:**
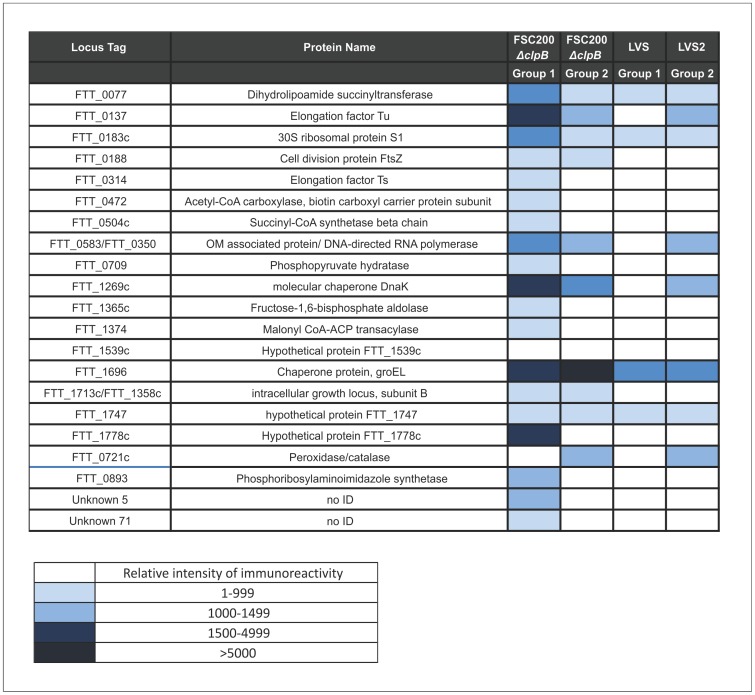
Immunoreactivity of sera from BALB/c mice after vaccination with FSC200Δ*clpB* or LVS. Mice were bled 28 days after immunization with 10^5^ CFU of FSC200*ΔclpB* or LVS. Each pool contained sera from five mice. Western blots were developed and scanned, and immunoreactive areas recorded as relative immunoreactivity, measured by densitometry. Shown are the top ranked immunodominant proteins. The identity of each immunoreactive area was determined by alignment with an equivalent protein stained gel, and immunoreactive spots identified by nLC-MSMS of their tryptic digests.

Mice immunized ID with 10^5^ CFU of FSC200*ΔclpB* or LVS were bled four weeks later, and serum from each group was pooled separately and used to probe 2DE Western blots of a whole cell lysate of SCHU S4. The identified immunodominant proteins are listed in figure 6. No reactivity was observed with pre-immune sera. Some differences in the reactivity pattern were observed between the pools of sera from mice immunised with LVS vs FSC200*ΔclpB*. Immunoreactive proteins included dihydrolipoamide succinyltransferase (FTT_0077), 30S ribosomal protein S1 (FTT_0183), chaperonin protein, GroEL (FTT_1696) and hypothetical protein FTT_1747. One of the serum pools from mice immunised with FSC200*ΔclpB*, recognised nine proteins that were not reactive at detectable levels with sera from LVS vaccinated animals. These proteins included three unknown proteins,and Elongation factor Ts (FTT_0314), Succinyl CoA (FTT_0504), phosphopyruvate hydratase (FTT_0709), Fructose-1,6-bisphosphate aldolase (FTT_1365), Malonyl CoA-ACP transacylase (FTT_1374) and hypothetical protein FTT_1778c). Finally, pooled sera from FSC200*ΔclpB* immunised mice showed between 2–3 fold greater total intensity of immunoreactivity, compared with sera from LVS vaccinated animals.

### Efficacy and Safety of FSC200 clpB Versus LVS or SCHUS4ΔclpB

**Figure 7:**
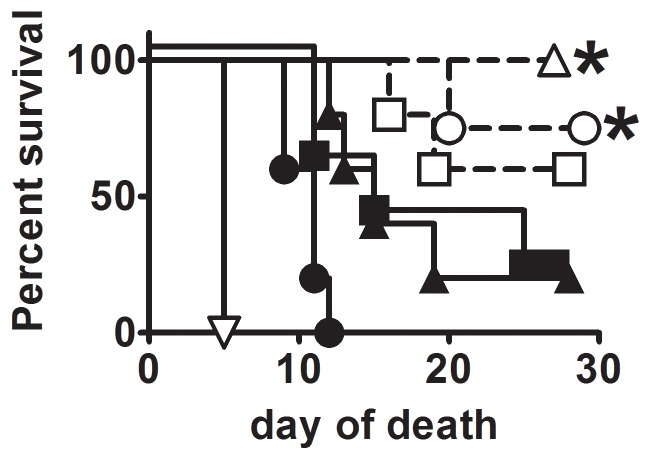
Efficacy comparison of SCHU S4 *ΔclpB* versus FSC200 *ΔclpB.* BALB/c mice (n = 5/group) were immunized with 10^3^ (squares), 10^5^ (circles), or 10^7^ (triangles) CFU of SCHU S4*ΔclpB* (open symbols) or FSC200*ΔclpB* (closed symbols). Immunized mice and naïve controls (inverted triangle) were challenged six weeks later IN with 105 CFU of SCHU S4. *, significantly better survival versus mice immunized with FSC200*ΔclpB*.

**Figure 8:**
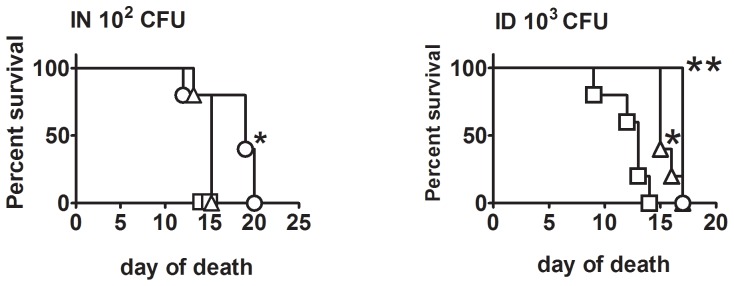
Virulence of vaccine strains for SCID mice. SCID mice were challenged ID or IN with SCHUS4*ΔclpB* (circle), FSC200Δ*clpB* (square), or LVS (triangle) and monitored for survival. *, significantly increased survival versus FSC200*ΔclpB*; **, significantly increased survival versus other strains.

To determine whether FSC200Δ*clpB* elicits a similar level of protection as SCHU S4Δ*clpB*, BALB/c mice were immunized ID with 10^3^, 10^5^, or 10^7^ CFU of one or other mutant, then challenged 6 weeks later IN with 105 CFU of SCHU S4 (figure 7). SCHU S4Δ*clpB* was more protective than FSC200Δ*clpB* at all test inocula, significantly so at doses of 10^5^ and 10^7^ CFU. Next, we assessed the residual virulence of SCHU S4Δ*clpB*, FSC200Δ*clpB* and LVS in SCID mice via ID and IN routes (figure 8) By the ID route, FSC200Δ*clpB* was significantly more virulent than LVS which, in turn, was significantly more virulent than SCHUS4Δ*clpB*. Relative virulence corresponded with bacterial burden (Table S1 in File S1). By the IN route FSC200*ΔclpB* was significantly more virulent for SCID mice than SCHU S4Δ*clpB.*

## Discussion

Cell-mediated immunity is required for protection against *F. tularensis* subsp. *tularensis* [21]. A single vaccination by scarification with *F. tularensis* LVS elicits essentially life-long cell-mediated immunity in humans, whereas humoral immunity disappears within ten years [22], [23]. However, human volunteer studies conducted during the 1960s indicated that the protective immunity provided by LVS against aerosol challenge with subsp. *tularensis* strain SCHU S4, waned within 6–12 months [5], [6], [24]. The anti-LVS antibody response was the sole immunological parameter assessed by the latter studies, and did not correlate with protection [25]. Because of these findings and the uncertain pedigree of LVS, we and others have been searching for alternative vaccine candidates. The major indication for vaccination against tularemia is to prevent a mass-casualty outbreak caused by the deliberate release of an aerosol of subsp. *tularensis* either on the battlefield or on civilians. A vaccine that is rapidly effective after a single dose would be preferable to combat both of the aforementioned scenarios. To this end, we developed a deletion mutant of SCHU S4 missing the gene for the chaperonin protein, clpB. It is much more effective and safer than LVS in a murine model of respiratory challenge with SCHU S4 [12]–[15]. Attempts to delete a second virulence gene from SCHU S4*ΔclpB* have resulted in almost complete loss of protective properties even in situations where the absence the other virulence gene alone causes only minor loss of virulence (Table S2 in File S1). For instance, 10 CFU of SCHU S4 *ΔrelA* kills mice more slowly than the same sized inoculum of the wild-type, but SCHU S4*ΔclpBΔrelA* elicits no protection against respiratory challenge with SCHU S4. This suggests that SCHU S4*ΔclpB* has reached the limits of its attenuation for efficacy purposes. Others have encountered a similar problem when combining otherwise highly protective single mutations in SCHU S4 [10].

In our hands, SCHU S4Δ*clpB* does not regain any measurable virulence or lose protective immunogenicity following multiple *in vivo* passages in mice, or when co-cultured multiple times with SCHU S4 Δ*iglC*, another highly attenuated mutant that possesses an intact *clpB* gene (data not shown). This indicates that the *clpB* deletion mutation is essentially irreversible. Nevertheless, a similar mutation on a subsp. *holarctica* background could be as effective as and potentially safer than SCHU S4 *ΔclpB*. The current study shows that this is not the case on either count. Subsp. *holarctica* strain FSC 200 is highly virulent for mice, and deleting its *clpB* gene results in severe attenuation. Like SCHU S4*ΔclpB*, FSC200*ΔclpB* persists longer and at higher levels in mice than LVS after ID inoculation, suggesting that the former might be more virulent. However, by other measures, namely IN LD_50_ and skin reactogenicity, both *ΔclpB* mutants are less virulent than LVS. FSC200*ΔclpB* elicits greater protection than LVS against respiratory challenge with SCHU S4, and this is associated with the production of significantly higher levels of IL-17 in the lungs one week following challenge with virulent SCHU S4. This represents the third distinct scenario involving *ΔclpB* mutants of *F. tularensis* in which we have observed this association between protection and late onset production of pulmonary IL-17. Previously, we have seen this phenomenon when comparing the superior efficacy of SCHU*ΔclpB* versus LVS in BALB/c mice, and when comparing the superior efficacy of the former in BALB/c *versus* C57BL/6 mice [13], [14]. In contrast, no significant differences were observed in pulmonary IFNγ or TNFα levels in any of these three situations, despite the fact that these two cytokines are known to be critical for vaccine-induced protection against pulmonary challenge with virulent *F. tularensis*. Therefore, it might be that IFNγ,TNFα,and IL-17 are all required to be present in appropriate amounts and at the appropriate time to ensure adequate protection against respiratory challenge with virulent bacteria. A similar phenomenon has been observed for live vaccine induced protection against *Yersinia pestis* where pluripotent CD4+ T cells secreting TNFα and IL-17 or TNFα, IFNγ and IL-17 appear to be critical for host defense [26]. These three cytokines alone or combined are also known to be involved in vaccine-induced immunity against other intracellular and extracellular pulmonary bacterial pathogens [27]–[30]. Given the plethora of actions of all three cytokines, additional experimentation rather than excessive speculation will be required to determine their coordinated roles in immune defense against pulmonary *F. tularensis*.

FSC200*ΔclpB* provides significantly inferior protection compared to SCHU S4*ΔclpB*. This supports our hypothesis that immune responses to antigens specific to subsp. *tularensis* contribute to protection [31]. Additionally, FSC200*ΔclpB* is more virulent than SCHU S4*ΔclpB* for SCID mice, whilst LVS displayed intermediate virulence. The reason for the enhanced virulence of FSC200*ΔclpB* for SCID mice is unclear, but suggests that clpB is a more critical virulence factor for subsp. *tularensis* than for subsp. *holarctica*. Additionally,the Δ*clpB* mutant of the LVS strain has previously been characterized and found to be essentially avirulent [32]. Presumably, this is due to the *a priori* marked attenuation of LVS. In our hands LVS*ΔclpB* elicits excellent protection against dermal challenge, but not respiratory challenge with SCHU S4 (figure S5 in File S1).

Mice are much more susceptible to *F. tularensis* than humans, and could, therefore, require a substantially more robust immune response for effective protection. In this case, either *ΔclpB* mutant might safely provide equally good protection in humans, though whether they would outperform LVS clinically remains to be determined, possibly by comparative testing in a non-human primate model of respiratory tularemia. In this regard, LVS has previously be shown to be effective in macaques [33], and several additional monkey models of tularemia have been recently published, but not yet used for vaccination studies [34]–[36].

## Supporting Information

File S1 **Figure S1.** Attenuation of FSC200*ΔclpB*. BALB/c mice (n = 5/group) were challenged ID with 10^7^ CFU of FSC200*ΔclpB* (circle) or 10 CFU of complemented FSC200*ΔclpB* (squares), and were monitored for survival. **Figure S2.** Intranasal virulence of FSC200ΔclpB versus LVS. BALB/c mice were inoculated IN with 10^4^ (open circle) or 10^5^ (closed circle) CFU of FSC200*ΔclpB* or 10^3^ (open square) or 10^4^ (closed square) CFU of LVS and were monitored for survival. *, significantly shorter survival than mice challenged with the same dose of FSC200*ΔclpB.* **Figure S3.** *In vivo* growth kinetics of FSC200*ΔclpB* vs LVS. BALB/c mice (n = 4/group) were immunized ID with ∼ 10^5^ CFU of FSC200ΔclpB (circle) or LVS (square). Mice were killed on days 2,4,7, and 14 for bacteriology. *, significantly higher burden vs LVS. Dashed line shows limit of detection. **Figure S4.** Skin reactogenicity score chart for *F. tularensis* strains. **Figure S5.** Protection against ID or IN challenge with SCHU S4 following immunization with SCHU S4*ΔclpB* versus LVS*ΔclpB*. BALB/c mice were immunized ID with ∼10^5^ CFU of SCHUS4*ΔclpB* or LVS*ΔclpB*. Immunized and control mice were challenged six weeks later with 1000 CFU ID or 40 CFU IN of SCHU S4 and were monitored for survival. *, significantly longer survival than control mice; **, significantly longer survival than mice immunized with LVS*ΔclpB*. **Table S1.** Growth of SCHU S4*ΔclpB*, FSC200*ΔclpB*, and LVS in the tissues of SCID mice. **Table S2.** Attenuation and immunogenicity profiles of selected SCHU S4 deletion mutants administered ID or IN to BALB/c mice.
(PDF)

## References

[1] Sjostedt A (2007) Tularemia: history, epidemiology, pathogen physiology, and clinical manifestations. Ann N Y Acad Sci 1105: 1–29. 17395726 10.1196/annals.1409.009

[2] Dennis DT, Inglesby TV, Henderson DA, Bartlett JG, Ascher MS, et al (2001) Tularemia as a biological weapon: medical and public health management. JAMA 285: 2763–2773. 11386933 10.1001/jama.285.21.2763

[3] Tigertt WD (1962) Soviet viable *Pasteurella tularensis* vaccines. A review of selected articles. Bacteriol Rev 26: 354–373. 13985026 10.1128/br.26.3.354-373.1962 PMC441156

[4] Eigelsbach HT, Downs CM (1961) Prophylactic effectiveness of live and killed tularemia vaccines. I. Production of vaccine and evaluation in the white mouse and guinea pig. J Immunol 87: 415–425. 13889609

[5] Saslaw S, Eigelsbach HT, Prior JA, Wilson HE, Carhart S (1961) Tularemia vaccine study. II. Respiratory challenge. Arch Intern Med 107: 702–714. 13746667 10.1001/archinte.1961.03620050068007

[6] Hornick RB, Eigelsbach HT (1966) Aerogenic immunization of man with live Tularemia vaccine. Bacteriol Rev 30: 532–538. 5917334 10.1128/br.30.3.532-538.1966 PMC378235

[7] El Sahly HM, Atmar RL, Patel SM, Wells JM, Cate T, et al (2009) Safety, reactogenicity and immunogenicity of *Francisella tularensis* live vaccine strain in humans. Vaccine 27: 4905–4911. 19567246 10.1016/j.vaccine.2009.06.036 PMC2726995

[8] Conlan JW (2011) Tularemia vaccines: recent developments and remaining hurdles. Future Microbiol 6: 391–405. 21526941 10.2217/fmb.11.22

[9] Qin A, Scott DW, Thompson JA, Mann BJ (2009) Identification of an essential *Francisella tularensis* subsp. *tularensis* virulence factor. Infect Immun 77: 152–161. 18981253 10.1128/IAI.01113-08 PMC2612291

[10] Rockx-Brouwer D, Chong A, Wehrly TD, Child R, Crane DD, et al (2012) Low Dose Vaccination with Attenuated *Francisella tularensis* Strain SchuS4 Mutants Protects against Tularemia Independent of the Route of Vaccination. PLoS One 7: e37752. 22662210 10.1371/journal.pone.0037752 PMC3360632

[11] Ireland PM, LeButt H, Thomas RM, Oyston PC (2011) A *Francisella tularensis* SCHU S4 mutant deficient in gamma-glutamyltransferase activity induces protective immunity: characterization of an attenuated vaccine candidate. Microbiology 157: 3172–3179. 21852349 10.1099/mic.0.052902-0

[12] Conlan JW, Shen H, Golovliov I, Zingmark C, Oyston PC, et al (2010) Differential ability of novel attenuated targeted deletion mutants of *Francisella tularensis* subspecies *tularensis* strain SCHU S4 to protect mice against aerosol challenge with virulent bacteria: effects of host background and route of immunization. Vaccine 28: 1824–1831. 20018266 10.1016/j.vaccine.2009.12.001 PMC2822029

[13] Shen H, Harris G, Chen W, Sjostedt A, Ryden P, et al (2010) Molecular immune responses to aerosol challenge with *Francisella tularensis* in mice inoculated with live vaccine candidates of varying efficacy. PLoS One 5: e13349. 20967278 10.1371/journal.pone.0013349 PMC2953512

[14] Twine S, Shen H, Harris G, Chen W, Sjostedt A, et al (2012) BALB/c mice, but not C57BL/6 mice immunized with a DeltaclpB mutant of *Francisella tularensis* subspecies *tularensis* are protected against respiratory challenge with wild-type bacteria: association of protection with post-vaccination and post-challenge immune responses. Vaccine 30: 3634–3645. 22484348 10.1016/j.vaccine.2012.03.036

[15] Ryden P, Twine S, Shen H, Harris G, Chen W, et al (2012) Correlates of protection following vaccination of mice with gene deletion mutants of *Francisella tularensis* subspecies *tularensis* strain, SCHU S4 that elicit varying degrees of immunity to systemic and respiratory challenge with wild-type bacteria. Mol Immunol 54: 58–67. 23201853 10.1016/j.molimm.2012.10.043

[16] Salomonsson E, Kuoppa K, Forslund AL, Zingmark C, Golovliov I, et al (2009) Reintroduction of two deleted virulence loci restores full virulence to the live vaccine strain of *Francisella tularensis* . Infect Immun 77: 3424–3431. 19506014 10.1128/IAI.00196-09 PMC2715654

[17] Conlan JW, Chen W, Shen H, Webb A, KuoLee R (2003) Experimental tularemia in mice challenged by aerosol or intradermally with virulent strains of *Francisella tularensis*: bacteriologic and histopathologic studies. Microb Pathog 34: 239–248. 12732472 10.1016/s0882-4010(03)00046-9

[18] Golovliov I, Sjostedt A, Mokrievich A, Pavlov V (2003) A method for allelic replacement in *Francisella tularensis* . FEMS Microbiol Lett 222: 273–280. 12770718 10.1016/S0378-1097(03)00313-6

[19] Lindgren H, Honn M, Golovlev I, Kadzhaev K, Conlan W, et al (2009) The 58-kilodalton major virulence factor of *Francisella tularensis* is required for efficient utilization of iron. Infect Immun 77: 4429–4436. 19651867 10.1128/IAI.00702-09 PMC2747937

[20] Pasetti MF, Cuberos L, Horn TL, Shearer JD, Matthews SJ, et al (2008) An improved *Francisella tularensis* live vaccine strain (LVS) is well tolerated and highly immunogenic when administered to rabbits in escalating doses using various immunization routes. Vaccine 26: 1773–1785. 18308432 10.1016/j.vaccine.2008.01.005 PMC2678717

[21] Cowley SC, Elkins KL (2011) Immunity to Francisella. Front Microbiol 2: 26. 21687418 10.3389/fmicb.2011.00026 PMC3109299

[22] Eneslatt K, Normark M, Bjork R, Rietz C, Zingmark C, et al (2012) Signatures of T cells as correlates of immunity to *Francisella tularensis* . PLoS One 7: e32367. 22412866 10.1371/journal.pone.0032367 PMC3295757

[23] Eneslatt K, Rietz C, Ryden P, Stoven S, House RV, et al (2011) Persistence of cell-mediated immunity three decades after vaccination with the live vaccine strain of *Francisella tularensis* . Eur J Immunol 41: 974–980. 21442618 10.1002/eji.201040923 PMC3516913

[24] McCrumb FR (1961) Aerosol Infection of Man with Pasteurella Tularensis. Bacteriol Rev 25: 262–267. 16350172 10.1128/br.25.3.262-267.1961 PMC441102

[25] Saslaw S, Carhart S (1961) Studies with tularemia vaccines in volunteers. III. Serologic aspects following intracutaneous or respiratory challenge in both vaccinated and nonvaccinated volunteers. Am J Med Sci 241: 689–699. 13746662

[26] Lin JS, Kummer LW, Szaba FM, Smiley ST (2011) IL-17 contributes to cell-mediated defense against pulmonary *Yersinia pestis* infection. J Immunol 186: 1675–1684. 21172869 10.4049/jimmunol.1003303 PMC3075555

[27] Moffitt KL, Gierahn TM, Lu YJ, Gouveia P, Alderson M, et al (2011) T(H)17-based vaccine design for prevention of *Streptococcus pneumoniae* colonization. Cell Host Microbe 9: 158–165. 21320698 10.1016/j.chom.2011.01.007 PMC3061323

[28] Priebe GP, Walsh RL, Cederroth TA, Kamei A, Coutinho-Sledge YS, et al (2008) IL-17 is a critical component of vaccine-induced protection against lung infection by lipopolysaccharide-heterologous strains of *Pseudomonas aeruginosa* . J Immunol 181: 4965–4975. 18802100 10.4049/jimmunol.181.7.4965 PMC2597098

[29] Khader SA, Bell GK, Pearl JE, Fountain JJ, Rangel-Moreno J, et al (2007) IL-23 and IL-17 in the establishment of protective pulmonary CD4+ T cell responses after vaccination and during *Mycobacterium tuberculosis* challenge. Nat Immunol 8: 369–377. 17351619 10.1038/ni1449

[30] Higgins SC, Jarnicki AG, Lavelle EC, Mills KH (2006) TLR4 mediates vaccine-induced protective cellular immunity to *Bordetella pertussis*: role of IL-17-producing T cells. J Immunol 177: 7980–7989. 17114471 10.4049/jimmunol.177.11.7980

[31] Twine S, Bystrom M, Chen W, Forsman M, Golovliov I, et al (2005) A mutant of *Francisella tularensis* strain SCHU S4 lacking the ability to express a 58-kilodalton protein is attenuated for virulence and is an effective live vaccine. Infect Immun 73: 8345–8352. 16299332 10.1128/IAI.73.12.8345-8352.2005 PMC1307091

[32] Meibom KL, Dubail I, Dupuis M, Barel M, Lenco J, et al (2008) The heat-shock protein ClpB of *Francisella tularensis* is involved in stress tolerance and is required for multiplication in target organs of infected mice. Mol Microbiol 67: 1384–1401. 18284578 10.1111/j.1365-2958.2008.06139.x

[33] Eigelsbach HT, Tulis JJ, Overholt EL, Griffith WR (1961) Aerogenic immunization of the monkey and guinea pig with live tularemia vaccine. Proc Soc Exp Biol Med 108: 732–734. 13889610 10.3181/00379727-108-27049

[34] Twenhafel NA, Alves DA, Purcell BK (2009) Pathology of inhalational *Francisella tularensis* spp. *tularensis* SCHU S4 infection in African green monkeys (*Chlorocebus aethiops*). Vet Pathol 46: 698–706. 19276059 10.1354/vp.08-VP-0302-T-AM

[35] Nelson M, Lever MS, Dean RE, Savage VL, Salguero FJ, et al (2010) Characterization of lethal inhalational infection with *Francisella tularensis* in the common marmoset (*Callithrix jacchus*). J Med Microbiol 59: 1107–1113. 20558585 10.1099/jmm.0.020669-0 PMC3052436

[36] Nelson M, Lever MS, Savage VL, Salguero FJ, Pearce PC, et al (2009) Establishment of lethal inhalational infection with *Francisella tularensis* (tularaemia) in the common marmoset (*Callithrix jacchus*). Int J Exp Pathol 90: 109–118. 19335549 10.1111/j.1365-2613.2008.00631.x PMC2676706

