# Supplementary material for: A ΔclpB Mutant of Francisella tularensis Subspecies holarctica Strain, FSC200, Is a More Effective Live Vaccine than F. tularensis LVS in a Mouse Respiratory Challenge Model of Tularemia
Source: PLoS One. 2013 Nov 13;8(11):e78671. doi: 10.1371/journal.pone.0078671 (PMC3827231; doi:10.1371/journal.pone.0078671)
Supplement: File S1 — Figure S1. Attenuation of FSC200ΔclpB. BALB/c mice (n = 5/group) were challenged ID with 107 CFU of FSC200ΔclpB (circle) or 10 CFU of complemented FSC200ΔclpB (squares), and were monitored for survival. Figure S2. Intranasal virulence of FSC200ΔclpB versus LVS. BALB/c mice were inoculated IN with 104 (open circle) or 105 (closed circle) CFU of FSC200ΔclpB or 103 (open square) or 104 (closed square) CFU of LVS and were monitored for survival. *, significantly shorter survival than mice challenged with the same dose of FSC200ΔclpB. Figure S3. In vivo growth kinetics of FSC200ΔclpB vs LVS. BALB/c mice (n = 4/group) were immunized ID with ∼ 105 CFU of FSC200ΔclpB (circle) or LVS (square). Mice were killed on days 2,4,7, and 14 for bacteriology. *, significantly higher burden vs LVS. Dashed line shows limit of detection. Figure S4. Skin reactogenicity score chart for F. tularensis strains. Figure S5. Protection against ID or IN challenge with SCHU S4 following immunization with SCHU S4ΔclpB versus LVSΔclpB. BALB/c mice were immunized ID with ∼105 CFU of SCHUS4ΔclpB or LVSΔclpB. Immunized and control mice were challenged six weeks later with 1000 CFU ID or 40 CFU IN of SCHU S4 and were monitored for survival. *, significantly longer survival than control mice; **, significantly longer survival than mice immunized with LVSΔclpB. Table S1. Growth of SCHU S4ΔclpB, FSC200ΔclpB, and LVS in the tissues of SCID mice. Table S2. Attenuation and immunogenicity profiles of selected SCHU S4 deletion mutants administered ID or IN to BALB/c mice. (PDF) [file pone.0078671.s001.pdf]

Figure S1

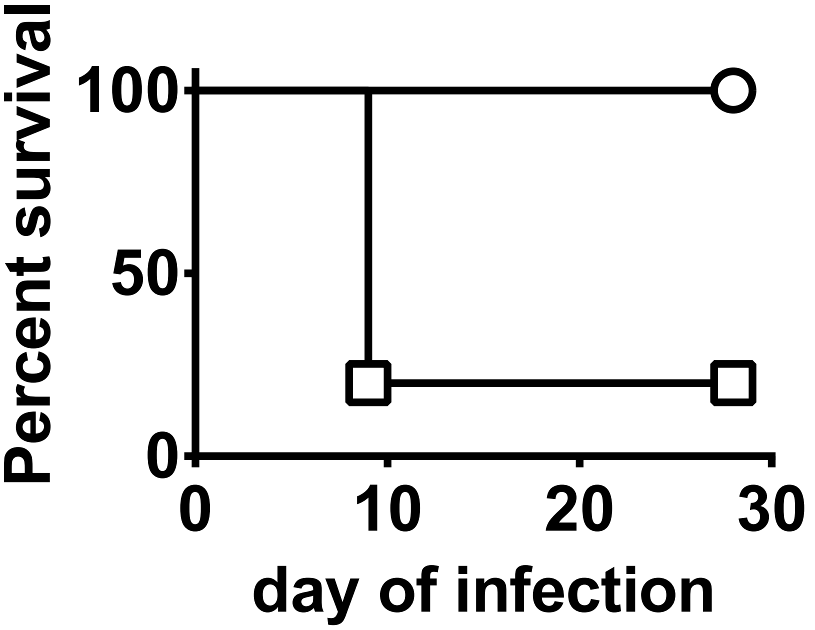

Figure S2

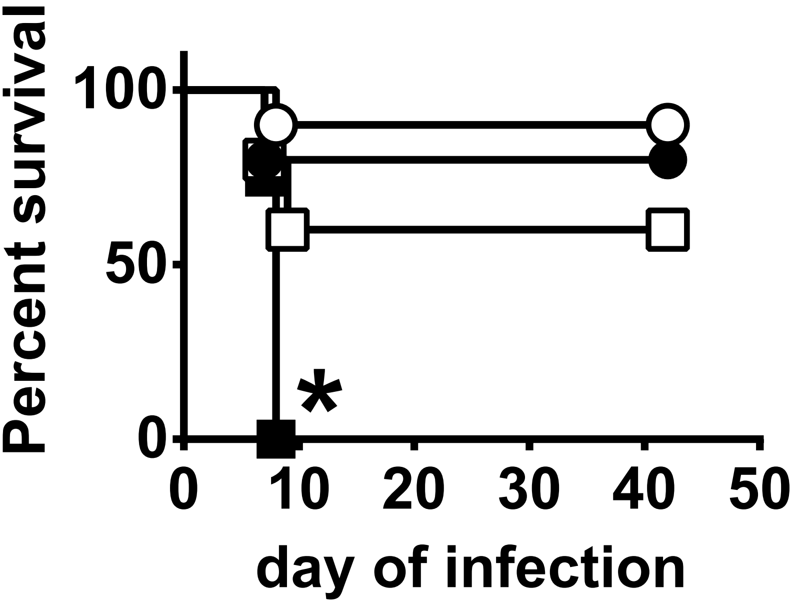

Figure S3

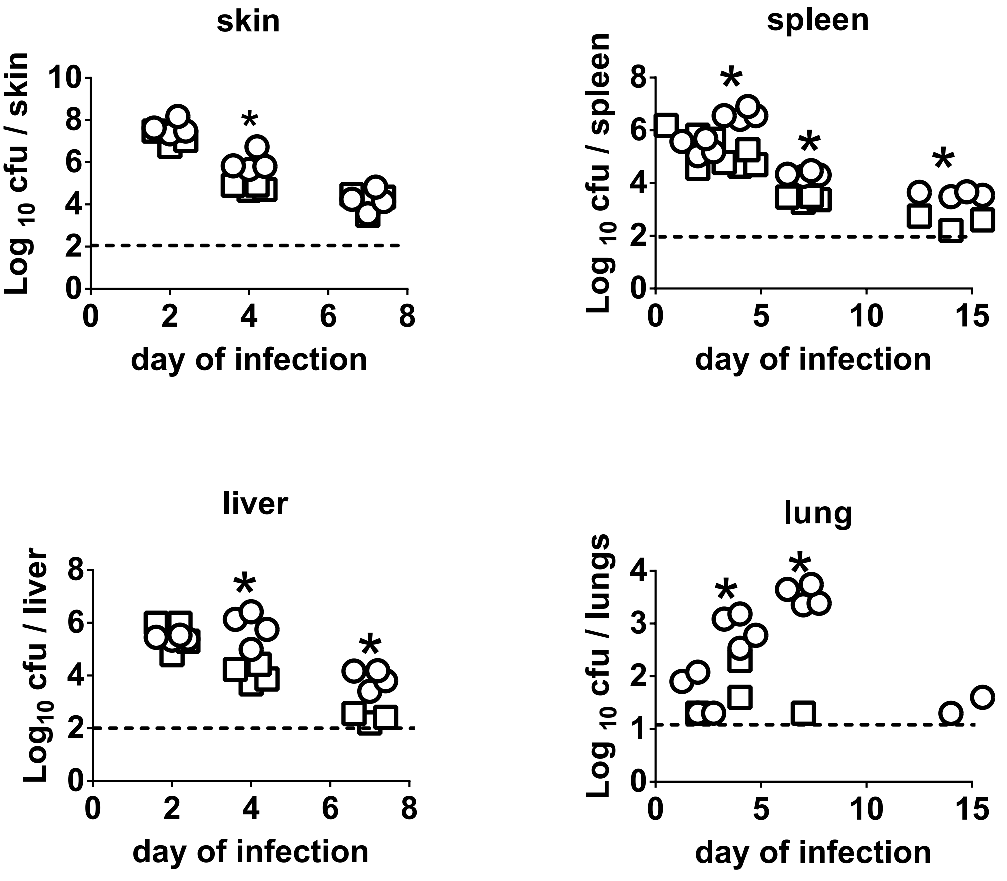

skin
Log 10 cfu / skin
day of infection
spleen
Log 10 cfu / spleen
day of infection
liver
Log10 cfu / liver
day of infection
lung
Log 10 cfu / lungs
day of infection

Figure S4

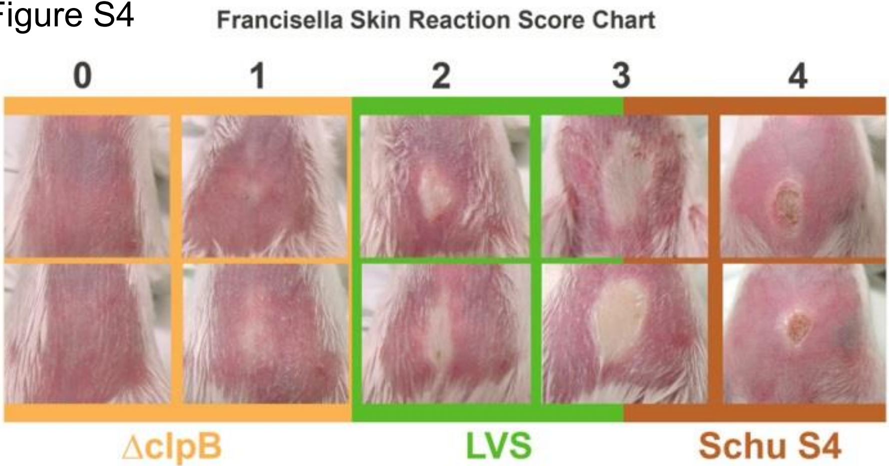

Francisella Skin Reaction Score Chart
0
1
2
3
4
ΔclpB
LVS
Schu S4

Figure S5

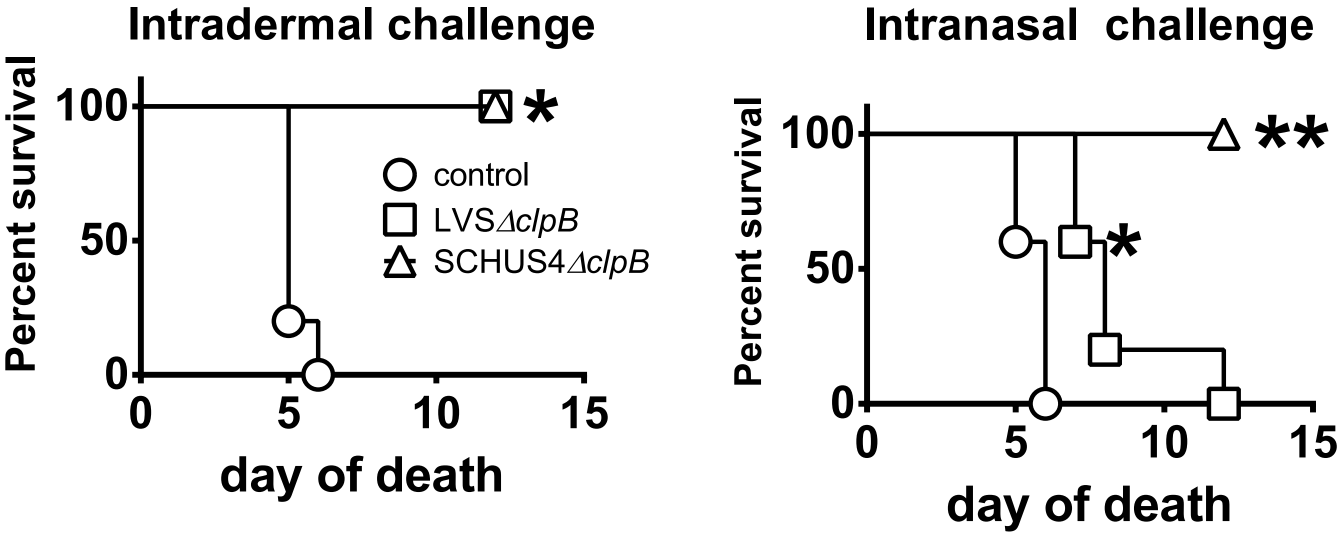

Intradermal challenge
Percent survival
100
50
0
0
5
10
15
day of death
control
LVSΔclpB
SCHUS4ΔclpB
*
Intranasal challenge
Percent survival
100
50
0
0
5
10
15
day of death
**
*

Table S1. Growth of attenuated strains of *F. tularensis* in SCID mice

	Log $_{10}$ CFU /organ ± SD				
	Lung	Liver	Skin	spleen	Blood
Bacteria					
SCHUS4*ΔclpB*	5.63 ±0.54	6.77 ±0.21*	6.15 ±0.37	6.47 ±0.13	4.13 ±0.37
FSC200*ΔclpB*	6.41± 0.92*	7.27 ±0.62*	7.37± 1.16 *	7.35± 0.36**	5.52± 0.97
LVS	5.14± 0.57	5.65± 0.51	5.35± 0.34	6.03± 0.41	4.42± 0.74

SCID mice (n=5 /group) were challenged ID with 10^3 CFU of one or other bacterial strain, and killed on day 8 of infection for bacteriology. *, significantly greater burden than LVS, **, significantly greater burden than SCHUS4*ΔclpB* or LVS

Table S2. Attenuation and immunogenicity profiles of selected SCHU S4 deletion mutants administered ID or IN to BALB/c mice.

Strain	ID LD_{50}	Protection against ID challenge[1]		Protection against aerosol challenge[1]	
		Vaccine Dose.	Protection	Vaccine Dose.	Protection
Δ*wbtC*	~10^7	10^3	++++	10^7	-
Δ*pmrA*	~10^1	10^1	++++	ND	ND
Δ*relA*	<10^1	ND	ND	ND	ND
Δ*fupA*	~10^4	10^3	++++	10^3	+
Δ*clpB*	>10^7	10^3, 10^5	++++	10^3,10^7	+++
Δ*ggt*	<10^7	10^3	++++	10^5	+++
Δ*capB*	<10^1	ND	ND	ND	ND
Δ*clpB*Δ*pmrA*	>10^7	ND	ND	10^7	++
Δ*clpB*Δ*relA*	>10^7	ND	ND	10^7	-
Δ*clpB*Δ*capB*	>10^7	10^7	++++	10^7	++
Δ*clpB*Δ*wbtC*	>10^7	10^7	++++	10^7	-
Δ*clpB*Δ*ggt*	>10^7	10^3	+++	10^7	+
Δ*clpB*Δ*fupA*	>10^7	10^7	++++	10^7	+
LVS	>10^7	10^3, 10^6	++++	10^3, 10^5, 10^6,10^7	+

Mice were challenged ≥ 4 weeks post-vaccination.

[1], the ID challenge dose was 1,000 SCHUS4 and the aerosol dose~ 100 CFU. All naïve mice died within 6 days of ID or IN challenge.

++++	all vaccinated mice survived challenge for >21 days
+++	at least 50% of vaccinated mice survived challenge for >21 days
++	≤ 20% vaccinated mice survived challenge for >21 days
+	mice survived significantly longer than naïve mice, but all died in <21 days
-	Mice did not survive significantly longer than naïve mice
ND	Not determined
